# Early infant prefrontal gray matter volume is associated with concurrent and future infant emotionality

**DOI:** 10.1038/s41398-023-02427-0

**Published:** 2023-04-17

**Authors:** Yicheng Zhang, Layla Banihashemi, Alyssa Samolyk, Megan Taylor, Gabrielle English, Vanessa J. Schmithorst, Vincent K. Lee, Amelia Versace, Richelle Stiffler, Haris Aslam, Ashok Panigrahy, Alison E. Hipwell, Mary L. Phillips

**Affiliations:** 1 University of Pittsburgh Swanson School of Engineering, Department of Bioengineering, Pittsburgh, PA USA; 2 University of Pittsburgh School of Medicine, Department of Psychiatry, Pittsburgh, PA USA; 3 UPMC Children’s Hospital of Pittsburgh, Department of Pediatric Radiology, Pittsburgh, PA USA

**Keywords:** Predictive markers, Psychiatric disorders

## Abstract

High levels of infant negative emotionality (NE) are associated with emotional and behavioral problems later in childhood. Identifying neural markers of high NE as well as low positive emotionality (PE) in infancy can provide neural markers to aid early identification of vulnerability, and inform interventions to help delay or even prevent psychiatric disorders before the manifestation of symptoms. Prefrontal cortical (PFC) subregions support the regulation of NE and PE, with each PFC subregion differentially specializing in distinct emotional regulation processes. Gray matter (GM) volume measures show good test-retest reliability, and thus have potential use as neural markers of NE and PE. Yet, while studies showed PFC GM structural abnormalities in adolescents and young adults with affective disorders, few studies examined how PFC subregional GM measures are associated with NE and PE in infancy. We aimed to identify relationships among GM in prefrontal cortical subregions at 3 months and caregiver report of infant NE and PE, covarying for infant age and gender and caregiver sociodemographic and clinical variables, in two independent samples at 3 months (Primary: *n* = 75; Replication sample: *n* = 40) and at 9 months (Primary: *n* = 44; Replication sample: *n* = 40). In the primary sample, greater 3-month medial superior frontal cortical volume was associated with higher infant 3-month NE (*p* < 0.05); greater 3-month ventrolateral prefrontal cortical volume predicted lower infant 9-month PE (*p* < 0.05), even after controlling for 3-month NE and PE. GM volume in other PFC subregions also predicted infant 3- and 9-month NE and PE, together with infant demographic factors, caregiver age, and/or caregiver affective instability and anxiety. These findings were replicated in the independent sample. To our knowledge, this is the first study to determine in primary and replication samples associations among infant PFC GM volumes and concurrent and prospective NE and PE, and identify promising, early markers of future psychopathology risk.

## Introduction

Childhood-onset disorders carry elevated personal, societal, and healthcare costs relative to disorders that emerge later in life [1–4] and often have a more persistent course and poorer treatment response [5, 6]. There is thus a critical need to identify neural markers of future psychopathology risk to aid early identification of vulnerability and inform interventions to help delay or even prevent these disorders prior to the manifestation of symptoms.

As early as infancy, there are behavioral indicators of future psychopathology risk. The threshold, magnitude, duration, and frequency of emotional arousal, or negative emotionality (NE) and positive emotionality (PE), can be evaluated within the first months of infancy. High levels of infant NE, the frequency, intensity, and duration of crying, predict emotional and behavioral problems in childhood, including increased risks of developing depression, anxiety, and behavioral disorders [7–11]. Although fewer studies have examined infant PE, the frequency and intensity of smiling and laughter, as a predictor for future emotional functioning, some evidence suggests that low PE in infancy is related to behavioral inhibition and depression in late childhood [12–15]. Yet, while these behavioral indices can predict future emotional and behavioral problems, neural markers of high NE and/or low PE in infancy provide early markers of risk prior to the manifestation of the disease that are more proximal to underlying neurodevelopment and neural mechanisms than behavioral measures of future risk. Thus, these neural markers can ultimately guide the development and neurodevelopmental timing, as well as monitor the effectiveness, of new interventions to help reduce such risk.

Prefrontal cortical (PFC) regions in several largescale neural networks support the regulation of NE and PE [16–18]. These networks include the default mode network (DMN) centered on the medial prefrontal cortex (mPFC)-posterior cingulate/posterior parietal cortex (precuneus) [19, 20]; the salience network (SN) centered on the caudal anterior cingulate cortex (cACC)-anterior insula [21]; the frontoparietal central executive network (CEN), centered on the dorsolateral prefrontal cortex (dlPFC), as well as the lateral posterior parietal cortex (lPPC) [21, 22]; and the ventral attention network (VAN), centered on the ventral frontal cortex (VFC)-temporoparietal junction (TPJ) [23]. The DMN supports self-referential processing [24, 25]; the SN detects the most contextually important information to guide behavior [25, 26]; the CEN supports planning and top-down inhibitory control processes [27]; and the VAN detects unexpected stimuli and supports reorientation of attention [23].

While it is well established that these largescale neural networks develop across infancy [28–31], childhood, and adulthood [32–36], few studies have examined how these networks support NE and PE in infancy. Gray matter (GM) measures show good test-retest reliability [37–39], are critical structural underpinnings of neural activity, and thus have potential use as neural markers of NE and PE that may also highlight future psychopathology risk. Additionally, GM volume measures show better test-retest reliability than cortical thickness measures, which are other widely used measures of GM structure [39]. Cortical surface area is another potential measure, but is less informative than GM cortical volume as this measure does not reflect the total amount of GM in a given region, while GM volume measures are composites of cortical thickness and surface area measures. However, no study has yet examined relationships among GM structural measures and NE and PE in infancy. Studies in adults have, however, shown GM volumetric abnormalities in individuals with affective disorders, in particular, lower GM volumes in CEN regions, and, to a lesser extent, larger GM volumes in DMN regions [40]. Furthermore, studies also report alterations in prefrontal GM volumes in individuals at high familial risk of affective disorders [41] and in individuals with first episode major depressive disorder [42], indicating that GM volume alterations might precede, and thus confer risk for, the onset of affective disorders.

Our goal was to identify relationships among GM in PFC regions in the above neural networks and infant measures of NE and PE examined concurrently and prospectively, as a first stage to identifying neural marker predictors of emotional dysregulation and mental health problems later in childhood and beyond. Findings indicate reductions in CEN regional GM volume in adults with affective disorders, and altered PFC GM volumes in individuals at risk for, and those in early stages of, affective disorders. While gray matter volume measures show good test-retest reliability, no studies to our knowledge examined relationships among prefrontal cortical GM and NE or PE in infancy. We, therefore, tested the overarching hypothesis that patterns of NE and PE associated with future psychopathology risk, i.e, high NE and low PE, would be associated with lower GM volume in CEN cortical regions and, potentially, greater GM volume in DMN and/or SN and VAN cortical regions. Concurrent relationships were modeled on 3-month infants as both the structural brain imaging and behavioral indices can reliably be measured at this age [43], while prospective behavioral indices were collected at 9 months, as neurodevelopment occurs rapidly during the 3-to-9-month period [44]. Independent replications were implemented to demonstrate the accuracy and stability of the trained models. As key sociodemographic and caregiving factors, including caregiver age, socioeconomic status, parental depression, affective instability, and anxiety are known to shape infant neurodevelopment [45–53], these were included as covariates.

## Methods

### Participants

All procedures in both primary and replication samples were approved by the University of Pittsburgh Human Research Protection Office. The primary sample of this study comprised 102 consented infant-caregiver dyads; recruitment and 9-month follow-up is ongoing. The participants were recruited from the local community, including the postnatal wards at the University of Pittsburgh Medical Center (UPMC) Magee-Womens Hospital, a practice-based research network Pediatric PittNet via the University of Pittsburgh Clinical and Translational Science Institute, and the Pitt+Me organization. The replication sample comprised 56 infant-caregiver pairs recruited from the population-based, longitudinal Pittsburgh Girls Study (PGS). Exclusion criteria for both samples were: (1) in the infant: preterm birth (<37 wks post gestational age), low birth weight (<5.5 lb), Apgar score <7 at 5 min after birth, abnormal brain morphometry (occipitofrontal circumference <32 cm), other physical health problems leading to extended hospitalization, and contraindications for MRI scans (having pacemakers, aneurysm clips, or non-removable ferromagnetic material implanted); (2) for the caregiver, <18 yrs, as unable to give informed written consent, prenatal or concurrent substance exposure (through obstetric records or self-report), and <2 hours/day care of the infant.

### Imaging data acquisition

3-month-old infants in both samples were scanned on a 3 T Siemens MAGNETOM Skyra MRI system (Siemens Healthcare AG, Erlangen, Germany) with a 32-channel head coil at Children’s Hospital of Pittsburgh (CHP) using the un-sedated feed-and-bundle approach [Supplement]. Structural MRI (sMRI, either T1 or T2) scans deemed fair or better quality (i.e., with no obvious concentric rings via visual inspection by two independent observers) were considered usable for image processing and analysis. 77 3-month-old infants in the primary sample and 40 in the replication sample had at least one usable sMRI scan [Table 1]. The primary cause of poor image quality was movement, as the infants were in natural sleep.

**Table 1:** Summary of infant-caregiver dyads characteristics for analyses.

	Primary sample		Replication sample	
	3-month	9-month	3-month	9-month
	Mean ± SD	Mean ± SD	Mean ± SD	Mean ± SD
Total infant-caregiver pairs	77	44	40	40
Usable 3-month scans, T1(T1 used in mix-modality)/T2 ^	62(20)/57	35(11)/33	26(11)/29	26(11)/29
Infant				
Age, wks	15.17 ± 2.99	45.32 ± 9.20	14.00 ± 2.63	38.95 ± 3.36
Biological sex, male/female	38/39	21/23	21/19	21/19
Caregiver				
Caregiver age	31.47 ± 4.99	--	22.25 ± 1.28	--
Total government assistance sum	1.21 ± 1.45	--	3.58 ± 1.22	--
EPDS depressed mood	6.08 ± 5.26	6.45 ± 4.79	6.28 ± 5.54	5.55 ± 4.82
PAI-BOR affective instability	4.83 ± 3.54	5.11 ± 3.92	6.45 ± 2.77	--
STAI state anxiety	30.70 ± 10.23	29.48 ± 9.19	31.38 ± 9.68	28.63 ± 6.75
STAI trait anxiety	35.92 ± 11.48	35.00 ± 10.06	34.95 ± 8.37	33.60 ± 7.01
Emotional outcomes				
IBQ NE	2.97 ± 0.60 (n = 75)	3.26 ± 0.71	3.09 ± 0.67	3.55 ± 0.85
IBQ PE	3.94 ± 1.27 (n = 76)	5.46 ± 0.65	4.92 ± 1.18	5.46 ± 1.03

^In mix-modality samples, where T2 is usable, T2 was used preferentially.

### Measures

The caregiver-rated Infant Behavior Questionnaire-Revised (IBQ) short form was used to assess infant positive and negative emotionality at 3 months and at 9 months [43]. Infant negative emotionality (our proxy for NE in this study) was calculated by the numerical average of IBQ Sadness, Distress to Limitations, Fear, and reverse coded Falling Reactivity/Rate of Recovery from Distress subscales; infant positive emotionality (our proxy for PE in this study) was calculated by the numerical average of IBQ Smiling/Laughter and High-Intensity Pleasure subscales. Sociodemographic and other clinical variables analyzed for this study included infant biological sex and age (in weeks) at 3 and 9 months; caregiver age (years), socioeconomic status via the sum of total government assistance received at 3 months; caregiver postpartum depressed mood using the Edinburgh Postnatal Depression Scale (EPDS) [54], affective lability using the Personality Assessment Inventory-Borderline Features Scale (PAI-BOR) [55], and state and trait anxiety via the Spielberger State-Trait Anxiety Inventory (STAI) [56] assessed at infant age 3 and 9 months.

For the primary sample, 77 participants with useable sMRI scans had the above 3-month variables and 75 or 76 out of 77 had concurrent NE or PE, respectively; 44 of them also had all 9-month measures. For the replication sample, all 40 participants with useable sMRI scans had those measures at both timepoints. Any missing data in either sample were imputed using the mode completer strategy: the value for a participant of a missing variable was filled with the most frequently-occurring value of the variable within the corresponding sample. Caregiver PAI-BOR data in the replication sample were not collected at 9 months, so values of these 9-month measures were assigned using the respective 3-month values [Table 1 and Fig. S1].

### Imaging processing

Bias-corrected and skull-stripped 3-month infant brain sMRI data underwent FAST segmentation with FMRIB Software Library (FSL) 6.0 toolbox to extract the entire GM mask [57]. All PFC GM subregions (regions of interest, ROIs) were segmented based on the Desikan-Killiany-Tourville (DKT) cortical parcellation protocol [58]. Due to the different sizes, morphology, and contrast of infants' brains, directly registering the adult DKT atlas can result in inaccuracies. Here, a pseudo-DKT gyral map was created from the adult DKT labels dilated by a 7x7x7 voxel kernel, and a cerebrospinal fluid (CSF) mask was applied to remove mislabeled pixels in the CSF resulting from dilation. The adult DKT imaging template was registered to the input 3-month infant T1 or T2 MRI in native space via the symmetric diffeomorphic approach in the ANTs toolbox [59]. The resulting DKT gyral map was propagated to the input image by the same transformation. The GM mask for the input image was applied to the registered DKT gyral map for parcellating the GM by cortical subregions. The details of the parcellation accuracy from our pipeline are included in the Supplemental Information [Table S1]. This cortical parcellation pipeline will be made available from the corresponding author upon reasonable request in the future.

### Data analysis

For each prefrontal cortical subregion (ROI), the GM feature examined was the proportional volume of the subregion relative to whole-brain (global) cortical GM volume: the absolute volume of each subregion was summed across hemispheres, divided by the global cortical GM volume and standardized to the range of 0 to 1. Using this volumetric measure, GM volumes in 10 PFC ROIs defined by the DKT atlas in each hemisphere, including the medial and lateral orbitofrontal cortex (mOFC and lOFC), the rostral and caudal anterior cingulate cortex (rACC and cACC), the medial superior frontal cortex (SFC), the pars opercularis, the pars orbitalis, the pars triangularis, and the rostral and caudal middle frontal cortex were modeled with IBQ NE or PE measured concurrently at 3 months or prospectively at 9 months. To reduce multicollinearity of GM measures, the pars opercularis, pars orbitalis, and pars triangularis were combined as the ventrolateral prefrontal cortex (vlPFC), and rostral and caudal middle frontal cortex were combined as the dorsolateral prefrontal cortex (dlPFC). These PFC subregional ROIs were demonstrated in Fig. S2 and their absolute volumes were listed in Table S2. For each PFC subregion, GM volume derived from either imaging acquisition (T1 and T2) was included (mixed-modality sample) to maximize the sample size. Specifically, GM volume measures extracted from T2 scans were preferred for participants with both T1 and T2 scans usable [Table 1]; for participants having usable T1 scans only, GM volume measures were derived from T1 scans, and a correction term for imaging modality was applied.

#### Multivariate models

We first examined GM volume relationships with concurrent and prospective infant NE and PE via multivariate models. The proportional GM volume of each PFC subregion, imaging modality, and sociodemographic and clinical variables [Table 2] were independent variables for multiple regression, with concurrent or prospective NE or PE as dependent variables in four models: NE (a) PFC GM volumes-concurrent (sociodemographic/clinical) variables to 3-month NE and (b) PFC GM volumes-concurrent (sociodemographic/clinical plus 3-month NE) and prospective variables to 9-month NE; PE (a) PFC GM volumes-concurrent variables to 3-month PE and (b) PFC GM volumes-concurrent (plus 3-month PE) and prospective variables to 9-month PE.

**Table 2:** Sociodemographic and other clinical variables.

Concurrent 3 month	*Multivariate and bivariate*: Imaging modality; 3-month age (wks), biological sex; caregiver age, total government assistance sum, 3-month EPDS depressed mood, 3-month PAI-BOR affective instability, 3-month STAI state anxiety, 3-month STAI trait
Prospective 9 month	*Bivariate*: (a). Same as concurrent 3-month
	*Bivariate*: (b). (a) + 3-month IBQ NE/PE
	*Multivariate and bivariate*: (c). (b) + 9-month age (wks); 9-month EPDS depressed mood, 9-month PAI-BOR affective instability, 9-month STAI state anxiety, 9-month STAI trait

A 3-level feature-selection approach was applied for each outcome independently:

1. Participants from the primary sample were randomly split into training and testing sets, of which the testing set comprised 30% of the full sample, and the remaining 70% were assigned as the training set, with the homogeneity of sociodemographic and clinical characteristics preserved as much as possible. Constrained second-order polynomial models included second-order terms that consisted only of interaction terms between each cortical GM subregional volume and sociodemographic/clinical variables, as well as all first-order independent variables mentioned above. An elastic net regularizer was applied to the models for the primary feature selection [Supplement]. The models were trained on the training data to maximize the coefficient of determination, *R*^2^, via tuning the shrinkage parameter λ and L1 ratio α. These analytic steps were coded in Python 3.8.2 with the scikit-learn 0.24.1 package [60], and additional statistical steps were conducted with SPSS version 27.0 (IBM Corp., Armonk, NY).
2. The non-zero terms were further feature-selected through backward (for concurrent models, criterion: the probability of F-to-remove >0.100) or forward (for prospective models, criterion: the probability of F-to-enter <0.050) multiple regression on all data from the primary sample.
3. Selected features were then corrected for multiple comparisons using bootstrapped 95% confidence interval (CI) for each outcome separately. This approach resampled the full primary sample through random sampling with replacement 1000 times and reported the 95% CI of the unstandardized coefficient. The features were considered significant when the bootstrap 95% CI did not include 0 [61].

The surviving first and second-order features were then used in multiple regression models without regularization terms as multivariate models to determine the magnitude of the differences between exact and predicted IBQ outcomes explained by the selected PFC GM volumes and sociodemographic/clinical variables, via root mean square error (RMSE).

#### Bivariate models

Our multivariate models were hypothesis-driven to demonstrate the predictive ability of PFC subregional volumes with sociodemographic and clinical variables for infant NE and PE. Secondary to the multivariate models, we used post hoc bivariate models, as data-driven, focused analyses to examine the unique contribution of PFC GM volumes to infant NE and PE at 3 and 9 months, for better interpretation of PFC GM volume-NE and PE associations. Bivariate analyses were thus conducted for all PFC GM ROIs, using independent linear regressions to determine how the volume of each PFC GM subregion was associated with infant NE and PE. Imaging modality, sociodemographic and clinical variables for both infant and caregiver were covariates [Table 2; Supplement] to account for the impact of the infant’s external environment. In order to adjust for different categories of sociodemographic, caregiver clinical, and 3-month infant NE and PE, we used three layers of covariates in the prospective models: (a) adjusted for concurrent sociodemographic/clinical variables only; (b) adjusted for concurrent sociodemographic/clinical variables plus 3-month NE or PE; (c) adjusted for concurrent and prospective sociodemographic/clinical variables plus 3-month NE or PE. For each outcome, PFC subregions that survived bootstrap 95% CI multiple testing were considered significant predictors of the relevant IBQ outcome.

The independent replication sample was tested for modeling accuracies. For multivariate models, independent variables collected and processed via the same method as the primary sample were examined using the feature-selected and coefficient-trained multivariate regression models from the primary sample to determine the prediction accuracy of IBQ outcomes via RMSE. Each model determined by the primary sample was tested twice, separately on GM variables extracted from T1- and T2-weighted sMRI scans of participants in the replication sample. This was to validate the robustness of prediction for either structural imaging modality. For bivariate models, the sociodemographic and clinical covariate-corrected PFC subregional GM volumes that had significant relationships with NE or PE in the bivariate models of the primary sample were included in independent linear regression models with concurrent or prospective NE or PE. Due to the small sample size and influence of possible outliers, the significance of the correlation was reported using the corresponding outcome stratified-sampled bootstrapped 95% CI. This approach diminishes the influence of extreme outliers, weighing them less heavily. Sampling bootstrapping subgroups thus yields a more accurate distribution of the outcome variables that corresponds to the outcome variables of the full replication sample.

## Results

### Primary sample

The primary features selected by the elastic net for all concurrent or prospective NE or PE variables are listed in Table S3.

#### Associations between PFC GM volume and infant NE

a) Concurrent NE

**Fig. 1:**
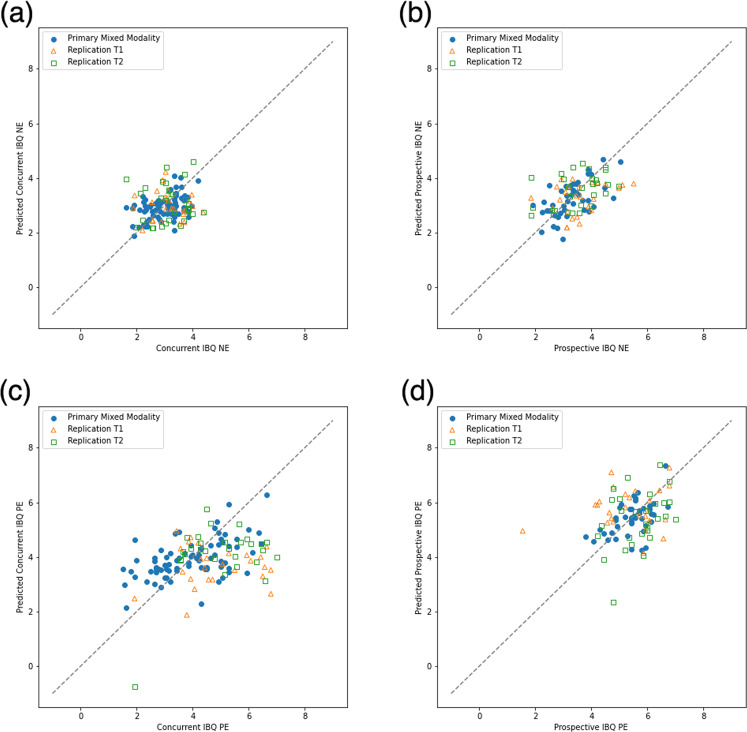
Multivariate actual-vs-predicted plots. **a** Concurrent NE; **b** Prospective NE; **c** Concurrent PE; **d** Prospective PE.

**Fig. 2:**
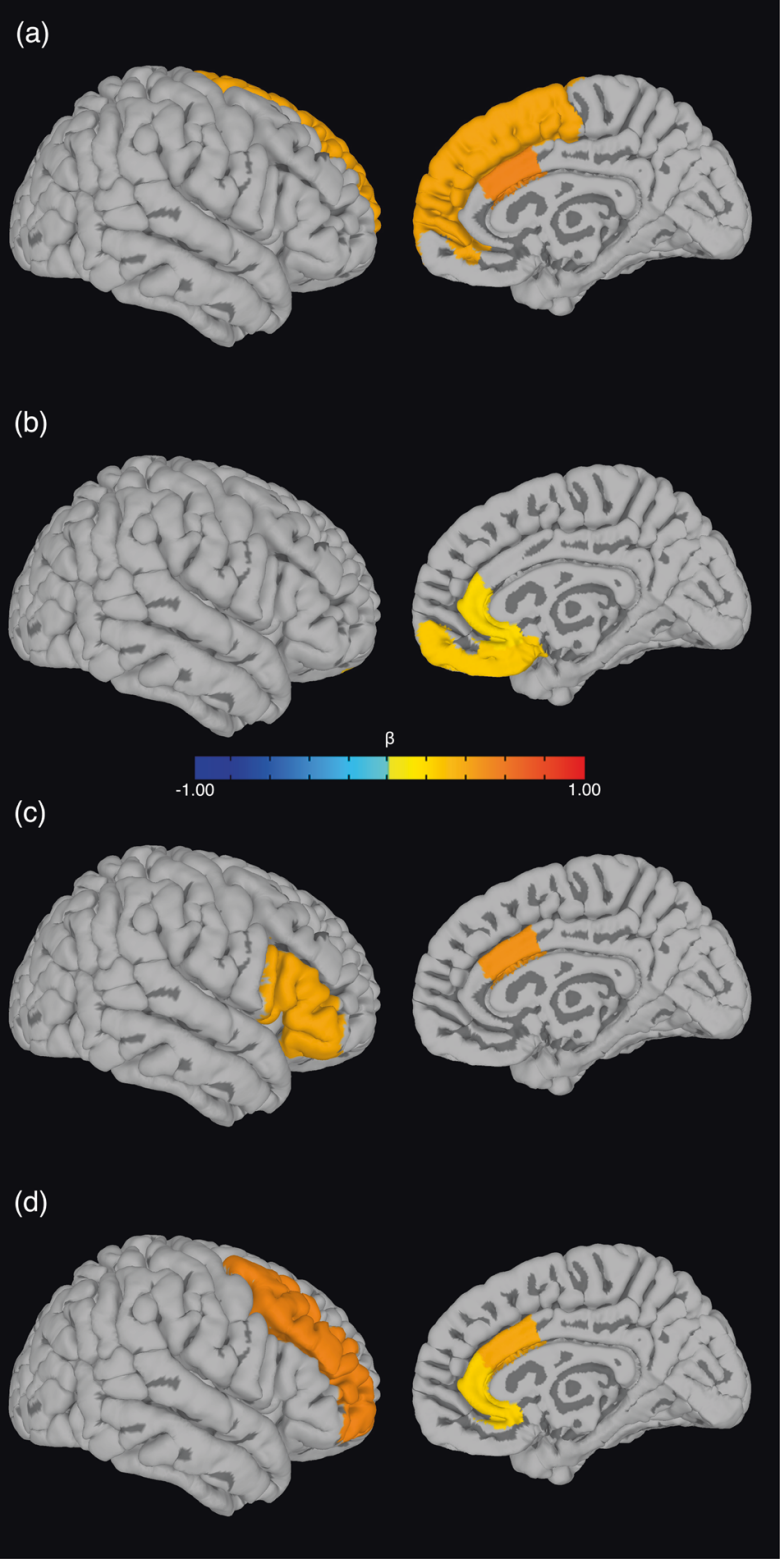
Significant (*p* < 0.05) first-order PFC subregions for multivariate models. **a** Concurrent IBQ NE: cACC, SFC; **b** Prospective IBQ NE: mOFC, rACC; **c** Concurrent IBQ PE: cACC, vlPFC; **d** Prospective IBQ PE: dlPFC, cACC, rACC. Left column: lateral; right column: medial.

**Fig. 3:**
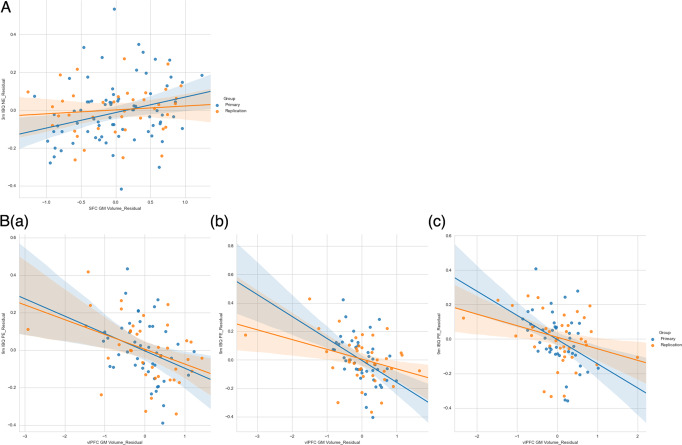
Covariate-corrected bivariate relationships between PFC GM volumes and infant emotional outcomes (solid lines as regression lines and shadowed areas as corresponding 95% confidence intervals). **A** Association between 3-month SFC GM volume and 3-month IBQ NE; **B** Associations between 3-month vlPFC GM volume and 9-month IBQ PE: **a** covaried by concurrent sociodemographic/clinical measures; **b** covaried by concurrent sociodemographic/clinical measures plus 3-month IBQ PE; **c** covaried by concurrent and prospective sociodemographic/clinical measures plus 3-month IBQ PE.

For the *multivariate model*, cACC GM volume (β = 0.533, *p* < 0.001), SFC GM volume (β = 0.402, *p* < 0.001); 3-month biological sex (β = 0.474, *p* = 0.001), 3-month caregiver STAI Trait anxiety (β = 0.112, *p* = 0.004); dlPFC GM volume × 3-month biological sex interaction (β = −0.246, *p* = 0.001), rACC GM volume × 3-month biological sex (β = −0.259, *p* = 0.013) were the final multiple-comparison-corrected significant features [Table S4]. The modeling accuracy for the mixed-modality primary sample was RMSE = 0.5685 [Fig. 1a; see Fig. 2a for the first-order relationships]. For the *bivariate models*, there was a significant positive correlation between SFC GM volume and concurrent infant NE ([Fig. 3A]; β = 0.254, *p* = 0.028; bootstrapped *p* = 0.018, 95% CI = [0.014, 0.135]).

b) Prospective NE

For the *multivariate model*, mOFC GM volume (β = 0.257, *p* = 0.007), rACC GM volume (β = 0.193, *p* = 0.016); 3-month caregiver PAI-BOR (β = 0.165, *p* = 0.002), caregiver age (β = 0.219, *p* = 0.007); dlPFC GM volume × 3-month NE (β = 0.240, *p* < 0.001) were the multiple-comparison-corrected significant features [Table S4]. The modeling accuracy for the mixed-modality primary sample was RMSE = 0.6111 [Fig. 1b; see Fig. 2b for the first-order relationships]. For the *bivariate models*, no covariate-corrected GM volume of the PFC subregion had a significant correlation with prospective infant NE.

#### Associations between PFC GM volume and infant PE

a) Concurrent PE

For the *multivariate model*, cACC GM volume (β = 0.477, *p* < 0.001), vlPFC GM volume (β = 0.396, *p* < 0.001); 3-month age (β = 0.496, *p* = 0.001), 3-month caregiver PAI-BOR (β = 0.375, *p* = 0.002); SFC GM volume × 3-month caregiver STAI state anxiety (β = 0.465, *p* < 0.001), vlPFC GM volume × 3-month caregiver STAI state anxiety (β = −0.448, *p* < 0.001), SFC GM volume × 3-month caregiver PAI-BOR (β = −0.408, *p* = 0.001), rACC GM volume × 3-month age (β = −0.385, *p* = 0.009) were the multiple-comparison-corrected significant features [Table S4]. The modeling accuracy for the mixed-modality primary sample was RMSE = 1.0755 [Fig. 1c; see Fig. 2c for the first-order relationships]. For the *bivariate models*, no covariate-corrected GM volume of the PFC subregion had a significant correlation with concurrent infant PE.

b) Prospective PE

For the *multivariate model*, dlPFC GM volume (β = 0.536, *p* < 0.001), cACC GM volume (β = 0.398, *p* < 0.001), rACC GM volume (β = 0.219, *p* = 0.023); 3-month PE (β = 0.902, *p* < 0.001); dlPFC GM volume × 3-month PE (β = −0.470, *p* < 0.001), cACC GM volume × 3-month PE (β = −0.425, *p* < 0.001), vlPFC GM volume × 3-month caregiver PAI-BOR (β = −0.084, *p* = 0.023), rACC GM volume × caregiver age (β = −0.106, *p* = 0.047) were the multiple-comparison-corrected significant features [Table S4]. The modeling accuracy for the mixed-modality primary sample was RMSE = 0.6319 [Fig. 1d; see Fig. 2d for the first-order relationships]. For the *bivariate models*, there were significant negative correlations between vlPFC GM volume and prospective infant PE with all three layers of covariates [Fig. 3B]: (a) 3-month covariates (β = −0.329, *p* = 0.029; bootstrapped *p* = 0.019, 95% CI = [−0.193, −0.039]); (b) 3-month covariates with 3-month PE (β = −0.465, *p* = 0.001; bootstrapped *p* = 0.001, 95% CI = [−0.256, −0.084]); (c) 3- and 9-month covariates with 3-month PE (β = −0.419, *p* = 0.005; bootstrapped *p* = 0.004, 95% CI = [−0.232, −0.057]).

### Replication sample

For the *multivariate models* [Fig. 1], the modeling accuracies on the replication sample were: NE (a) concurrent NE T1 RMSE = 0.8262, T2 RMSE = 0.9416; (b) prospective NE T1 RMSE = 0.8836, T2 RMSE = 0.7863; PE (a) concurrent PE T1 RMSE = 1.8474, T2 RMSE = 1.5332; (b) prospective PE T1 RMSE = 1.3103, T2 RMSE = 1.0822. For the *bivariate models* [Fig. 2], SFC GM volume corrected for concurrent covariates was positively correlated with concurrent infant NE (β = 0.097; bootstrapped *p* = 0.001, 95% CI = [0.014, 0.025]); vlPFC GM volume corrected for each layer of covariates [Table 2] was negatively correlated with prospective infant PE ((a) 3-month covariates: β = −0.419; bootstrapped *p* = 0.001, 95% CI = [−0.095, −0.065]; (b) 3-month covariates with 3-month PE: β = −0.387; bootstrapped *p* = 0.001, 95% CI = [−0.090, −0.059]; (c) 3- and 9-month covariates with 3-month PE: β = −0.314; bootstrapped *p* = 0.001, 95% CI = [−0.079, −0.050]).

## Discussion

The goal of this study was to identify PFC GM volumetric markers of early infant negative and positive emotionality, that may reflect neural mechanisms underlying the development of infant emotionality and predict risk of future psychopathology later in childhood and beyond. Our in-house cortical parcellation pipeline made it possible to reliably extract GM volumes in different PFC subregions from either T1- or T2-weighted 3-month infant brain sMRI data. We used two-model approaches to model and interpret the relationships between PFC subregional GM volumes and infant NE or PE. We observed a positive association between 3-month SFC GM volume and concurrent (3-month) NE, and a negative association between 3-month vlPFC volume and prospective (9-month) PE. These findings were also validated with an independent replication sample, demonstrating the stability of the findings from the primary sample.

Our finding that 3-month SFC GM volume was positively associated with concurrent NE provides some support for our hypothesis that greater DMN GM volume would be associated with greater NE, as the SFC is a cortical midline prefrontal cortical component of the DMN that is implicated in the processing of self-related information and evaluating others’ mental states [62, 63]. Furthermore, while the SFC is also involved in other functions, including cognitive control and motor functions, the anteromedial part of the SFC, in particular, has robust connectivity with other DMN regions [64]. Our finding is also consistent with previous reports of greater cortical thickness in the left rostral SFC in adolescents with anxiety disorders [65]. Additionally, in a young adult study, greater right rostral SFC GM volume was observed in social anxiety disorder patients compared with healthy controls [66]. Thus, greater SFC GM volume is a promising neural marker of higher early infant NE.

Our finding that 3-month vlPFC GM volume was negatively associated with prospective PE at 9 months demonstrates the potential of PFC GM indices to predict lower future infant PE, which could be a risk factor for later depression [12–15]. At the network level, the vlPFC is a key region in the VAN [23] and SN [67], together supporting attention to salient and unexpected environmental stimuli. This finding is in accord with our hypothesis, and parallels reports of greater cortical GM to whole tissue volume ratio in PFC subregions in the SN (bilateral cACC and left rACC) in male adolescents with callous-unemotional traits [68], greater cortical thickness of the SN (left cACC) in adolescents with major depressive disorder versus healthy controls [69], and greater cortical thickness of the left pars opercularis in the vlPFC in young adults with the major depressive disorder [70]. One interpretation of these findings is that greater VAN and SN GM volumes in early infancy promote greater attention toward emotionally-salient external stimuli, which, in turn, might predispose to compensatory over-regulation of emotion later in infancy. This, in turn, could result in lower PE—associated with behavioral inhibition [12, 14] and indicative of over-regulation of emotion—in later rather than early infancy. This also accords with the slower development of the SN than several other networks during the first year [71], such that the SN contributes to the development of emotional regulation capacity later in infancy, as cognitive abilities such as memory and arousal awareness increase [44]. Models using all 3 layers of covariates showed significant correlations, indicating that 3-month vlPFC GM volume predicted 9-month PE even when controlling for external factors and 3-month PE.

In addition to the significant GM volume predictors of early emotional behaviors indicated by the bivariate models, concurrent and prospective NE and PE were predicted by multivariate models with high accuracy. In the concurrent NE multivariate model, the significant PFC subregion of the corresponding bivariate model, i.e., SFC GM volume, also survived multiple steps of feature selection and multiple-comparison. In the prospective PE model, there was a complex combination of independent variables. While vlPFC GM volume was not a significant first-order predictor, it remained a significant predictor in the second-order model, with interaction with 3-month caregiver affective instability. This negative correlation indicated that greater vlPFC GM volume in infants with a caregiver with greater affective instability at 3 months predicted lower infant 9-month PE, supporting previous findings of worse emotional functioning in infants with psychiatrically-unwell mothers [72]. Other GM volumetric variables selected by the multivariate models were also potential predictors of infant NE and PE. In the concurrent NE model, positive associations between cACC GM volumes and NE supported our hypothesis that greater SN GM volume would be associated with higher infant NE. In the prospective NE model, positive associations among mOFC and rACC GM volumes and NE supported our hypothesis that greater DMN and SN GM volumes would be associated with higher infant NE, suggesting more sustained relationships among greater DMN and SN GM volumes and NE from 3 to 9 months. In the prospective PE model, positive associations between dlPFC GM volumes and PE supported our hypothesis that greater CEN volume would be associated with higher infant PE. There were also correlations among PFC GM subregions in different networks and PE outcomes that contradicted our hypotheses both concurrently and prospectively, including greater GM volume of cACC in SN and vlPFC in VAN with higher concurrent PE, and greater GM volume of cACC and rACC in SN with higher prospective PE. These inconsistent findings may be due to the nature of multivariate regression, in which additional higher-order predictors may suppress, neutralize, or even override, correlations observed in bivariate models. Overall, however, first-order subregional PFC GM volumetric findings from the multivariate models largely supported our hypothesis.

There were several relationships among sociodemographic/clinical variables and infant NE and PE in the multivariate models. Higher levels of caregiver-reported NE among male compared to female infants at 3 months, together with associations between greater caregiver trait anxiety at 3 months and higher infant NE concurrently, converge with studies showing that male infants are more vulnerable to maternal mental illness [49] and demonstrating relationships between caregiver anxiety and difficult infant temperament [46]. Greater caregiver affective instability at 3 months was associated with higher infant NE prospectively, paralleling previous reports of significant positive correlations between 3-month caregiver depression and 6-month infant NE [73]. Caregiver affective instability, in particular, impacts the development of infant emotional regulation, resulting in higher levels of infant NE [50–53], likely via less secure caregiver-child attachment and/or high levels of caregiver-expressed emotion or family conflict [74–76]. Greater caregiver age was associated with higher infant NE at 9 months, a finding that needs exploration in future studies. Greater infant age at 3 months was associated with higher PE concurrently, paralleling the developmental trajectory of infant PE [77]. Unexpectedly, greater caregiver affective instability at 3 months was associated with greater concurrent infant PE. While low PE in infancy is related to behavioral inhibition and depression in late childhood [12–15], high levels of PE are associated with externalizing psychopathology in childhood and adolescence [78–80]. Thus, caregiver affective instability might predispose to both high levels of infant NE and low and high levels of PE, associated with internalizing and externalizing psychopathology, respectively, later in child development. 3-month PE was a significant positive predictor of 9-month PE, which is consistent with prior reports of correlations between 3- and 9-month IBQ Smiling/Laughter scores [81].

There were more interactions between GM volume in PFC subregions and caregiver affect and age variables underlying concurrent and prospective PE. Given that low and high levels of infant PE are each associated with future predisposition to psychopathology, as indicated above, while predominantly high levels of infant NE are associated with future internalizing psychopathology, it is likely that a greater number of caregiver factors shape the developing relationships between GM volumes and PE than NE in infancy, although there is no research to our knowledge comparing the influences of caregiver factors on relationships between developing GM and NE versus PE in infancy. Together, our findings indicate that (1) the combination of GM volume across different PFC subregions within networks, as well as independently in distinct PFC subregions, predict both infant NE and PE; (2) sociodemographic and clinical factors also impact infant NE and PE; and (3) PFC subregional GM volumes and sociodemographic and clinical variables together improve the prediction of prospective emotional behavior outcomes, especially PE.

The inconsistent findings from bivariate and multivariate models might have been due to the method of controlling for sociodemographic and clinical variables: in bivariate models, the volume of each PFC ROI was partially correlated with the outcomes by controlling the imaging modality, sociodemographic and clinical covariates; while in multivariate models, these variables were treated with the same priority as GM volume variables. Therefore, the possible impact of external environmental variables, which can also impact the maturation of the infant brain and likely contribute to changes in PFC GM volumes [82], were examined in association with GM volume measures in multivariate models.

The main limitation of this study was the sample size, especially for the prospective data. This is the first study to our knowledge to show associations among infant PFC GM volumes and emotional behaviors in 3-month-old infants, however; and we applied several layers of optimization to ensure the accuracy and stability of the findings. First, we used an in-house pipeline that is compatible for either T1- or T2-weighted sMRI data, to maximize the number of participants with usable GM volume data and included imaging modality as an independent variable in multivariate models and as a covariate in bivariate models. Second, we used bootstrapping to correct for multiple comparisons. Finally, and most critically, we used an independent replication sample to validate our findings for both multivariate and bivariate models. Our future work will use multimodal imaging approaches to examine relationships among indices of PFC structure and function and developing NE and PE in infancy and childhood.

We show, using multivariate and bivariate approaches, that network-wide key PFC GM volume measures and combinations of PFC GM volume measures, together with caregiver measures of emotional regulation in particular, are associated with infant NE and PE. Our approach provides neural markers of future psychopathology risk that reflect underlying neural mechanisms of infant NE and PE to not only inform future risk assessment, but to ultimately guide the development, timing, and monitoring of the effectiveness of new interventions to help reduce such risk.

## Supplementary information

Early Infant Prefrontal Gray Matter Volume is Associated with Concurrent and Future Infant Emotionality Supplemental Information
