# Supplementary material for: Early infant prefrontal gray matter volume is associated with concurrent and future infant emotionality
Source: Transl Psychiatry. 2023 Apr 17;13:125. doi: 10.1038/s41398-023-02427-0 (PMC10110602; doi:10.1038/s41398-023-02427-0)
Supplement: Supplementary file 1 — Early Infant Prefrontal Gray Matter Volume is Associated with Concurrent and Future Infant Emotionality Supplemental Information [file 41398_2023_2427_MOESM1_ESM.pdf]

Supplemental Information

MRI Scanning

3-month-old infants were scanned under the following imaging parameters: Primary sample: (1) Magnetization prepared rapid gradient echo (MP-RAGE) T1-weighted MRI: field of view (FOV)=205 mm, voxel dimensions=1.0x1.0x1.0 mm^3, TE/TR=3.3/2,400 ms, TI=1,200 ms, flip angle=8°; (2) 3D turbo spin echo (TSE) T2-weighted MRI: FOV=200 mm, voxel dimensions=0.8x0.8x0.8 mm^3, TE/TR=563/3,200 ms; Replication sample: (1) MP-RAGE T1-weighted MRI: FOV=256 mm, voxel dimensions=1.0x1.0x1.0 mm^3, TE/TR=3.16/2,400 ms, TI=1,200 ms, flip angle=8°; (2) 3D TSE T2-weighted MRI: FOV=180 mm, voxel dimensions=0.7x0.7x0.7 mm^3, TE/TR=420/3,100 ms.

Behavioral Measurements

The well-validated IBQ-R questionnaire consists of 14 scales (Activity Level, Distress to Limitations, Fear, Duration of Orienting, Smiling and Laughter, High Pleasure, Low Pleasure, Soothability, Falling Reactivity/Rate of Recovery from Distress, Cuddliness, Perceptual Sensitivity, Sadness, Approach, and Vocal Reactivity) with 91 questions indicating the frequency of various infant emotions and behaviors in the past 7 days. Each question is scored by a Likert-type scale, from 1 (=never) to 7 (=always), and each scale is scored by the arithmetic mean of the questions under the category.

To avoid redundant variables for the infant age at 3 months due to gaps between scanning and outcome measurement dates, 3-month IBQ measurements were taken within 2-3 weeks of the scheduled scans (mean 2.87±1.35 wks between).

Elastic Net Feature Selection

An elastic net regularizer, which combines both L1 and L2-norm term, was applied to the models for the primary feature selection. Regression with the L2-norm term, also known as ridge regression, can help solve the remaining multicollinearity problems by minimizing the coefficients; Regression with the L1-norm term, known as Least Absolute Shrinkage and Selection Operator (LASSO), can reduce the number of variables by assigning the coefficients to absolute zero. A combination of using both L1- and L2-penalty terms, i.e., elastic net, can benefit from both coefficient shrinkage by the L2-norm and variable selection by the L1-norm.

Bivariate Model Coefficients

Infant age and biological sex, caregiver age, total government assistance received, EPDS, PAI-BOR, STAI state and trait anxiety scores at 3 months, together with the imaging modality from which the GM volume measures were extracted, were 3-month covariates for modeling associations among GM volume measures and concurrent emotional outcomes. Three different covariate groups were used in models examining relationships among GM volume measures and prospective (9-month) outcomes. The first group used the same 3-month covariates as the concurrent models; the second group added the 3-month relevant IBQ outcomes; and the last group added infant age, caregiver EPDS, PAI-BOR, STAI state and trait anxiety scores at 9 months.

3-month Infant Imaging Cortical Parcellation Quality

To justify the registration quality of our pipeline, we used the manually labeled M-CRIB 2.0 T2 neonatal cortical parcellation atlas [1] as ground truth to calculate volumes and Dice Coefficients for each PFC subregion we used for this study (summarized in Table S1). We found that our automated parcellated GM volume values are largely comparable to the manually parcellated GM volume values and the labelling of corresponding PFC subregions overlaps.

Replacement of 9-month PAI-BOR Values in the Replication Sample

In the replication sample, the closest PAI-BOR was collected at 3 months. In the primary sample, 3-month PAI-BOR was highly correlated with 9-month PAI-BOR when we constrained the intercept to 0 ($p<0.001$; Figure S2). Therefore, we chose to use the replication sample's own 3-month PAI-BOR data instead of assigning average or random PAI-BOR values to represent 9-month measures.

Furthermore, according to our models trained on the primary sample's full 9-month PAI-BOR data, no 9-month multivariate models selected 9-month PAI-BOR as a significant feature for the prediction, and the presence or absence of synthetic replacement of the 9-month PAI-BOR in the replication sample did not affect the prediction accuracy of the replication sample. As for the bivariate models, the first 2 layers of covariates [Table 2, Prospective Bivariate (a) and (b), pg. 21] included only concurrent variables and would not be affected by the absence of synthetic replacement of the 9-month PAI-BOR in the replication sample. We ran the bivariate models again in both samples without the 9-month PAI-BOR as a covariate for the third layer of covariates [Table 2, Prospective Bivariate (c), pg. 21], and the correlations were $\beta=-0.437$, $p=0.003$ for the

primary sample and β=-0.314, p=0.049 for the replication sample, upholding similar significant correlations as the original bivariate model covaried by the concurrent and prospective factors.

References

1. Alexander B, Loh WY, Matthews LG, Murray AL, Adamson C, Beare R, et al., *Desikan-Killiany-Tourville atlas compatible Version of M-CRIB neonatal parcellated whole brain atlas: the M-CRIB 2.0.* Frontiers in Neuroscience, 2019. **13**: p. 34.

Figure S1. 3-to-9-month Caregiver PAI-BOR Affective Instability Relationships.

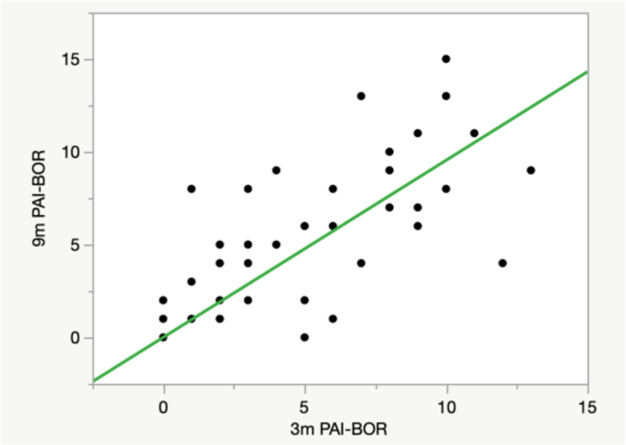

Figure S2. 3-month PFC Subregional GM ROIs. A. T1 images; B. T2 images. Left column: sagittal; middle column: coronal; right column: axial.

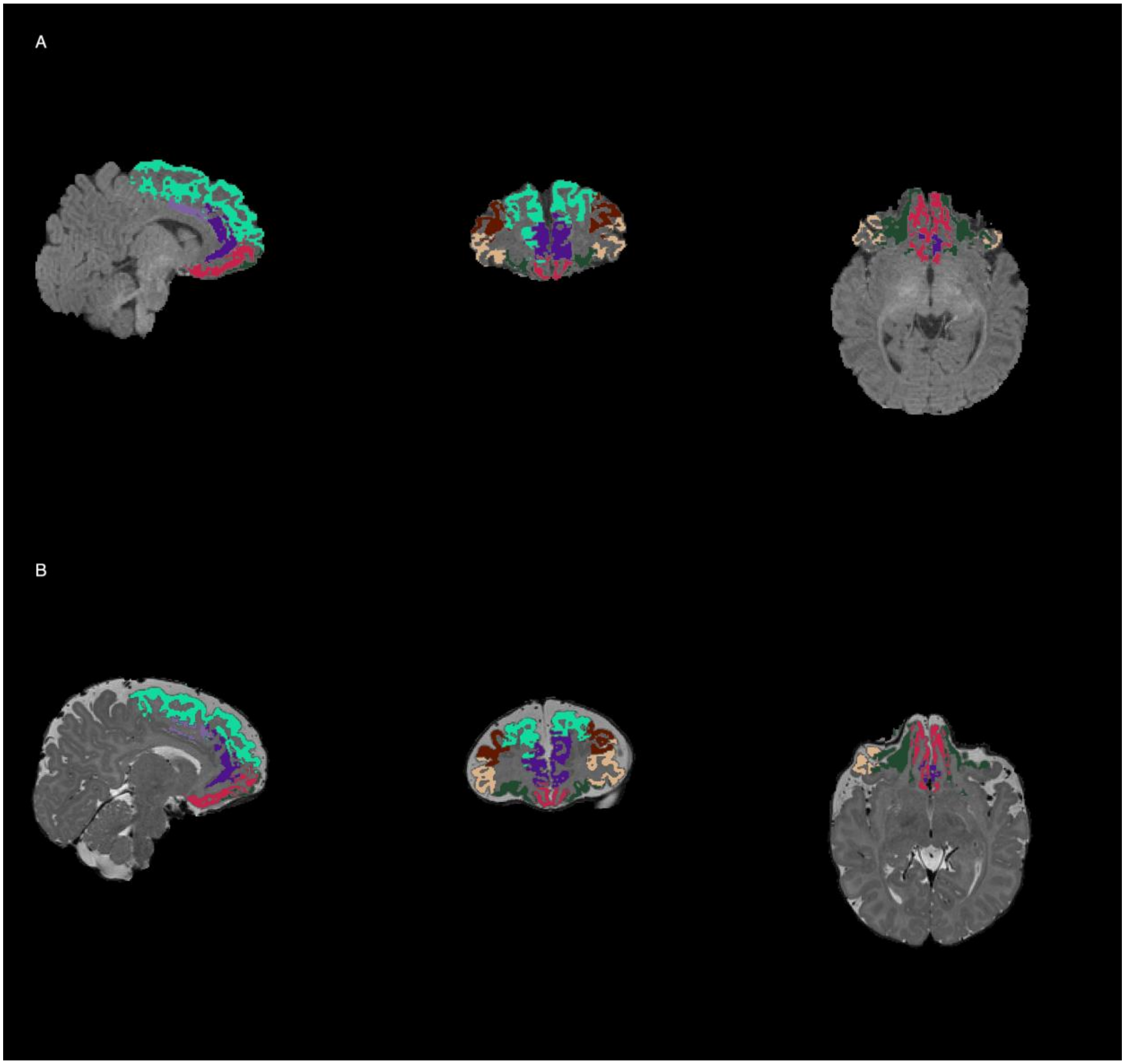

Table S1. Automated and Manual Cortical Parcellation Comparison. A. Mean volumes; B. Dice Coefficients.

A.

Mean Volume (mm^3)	*mOFC*	*lOFC*	*rACC*	*cACC*	*SFC*	*vlPFC*	*dlPFC*
Automated Left	5116.4	7317.5	7356.2	4300.5	27838.4	12235.3	18668.3
Manual Left	6621.1	9835.9	3529.1	3915.2	32126.2	16221.9	22392.7
Automated Right	6196.8	8562.9	5101.6	3878.3	35324.6	12507.3	17473.6
Manual Right	7119.1	10971.2	4351.2	4708.6	31774.9	15614.8	22358.2

B.

Dice (Mean±SD)	*mOFC*	*lOFC*	*rACC*	*cACC*	*SFC*	*vlPFC*	*dlPFC*
Left	0.61±0.06	0.75±0.02	0.42±0.11	0.53±0.10	0.75±0.04	0.73±0.04	0.75±0.03
Right	0.62±0.05	0.80±0.03	0.59±0.08	0.56±0.06	0.79±0.03	0.73±0.04	0.75±0.02

Table S2. Raw 3-month PFC Subregional GM Volume (Averaged by hemisphere)

	mOFC	*lOFC*	*rACC*	*cACC*	*SFC*	*vlPFC*	*dlPFC*
*1^*	2145	3368	2027	2073	16726	6035	9774
*2^*	2089	3327	1860	1481	15385	5929	9155
3	2422	3574	2363	1662	14479	6074	8361
*4^*	2256	3076	2397	1910	16987	5194	9596
*5^*	2335	3999	2266	1749	15647	6507	9682
*6^*	2189	3104	2705	1822	19372	5921	10246
7	2842	4161	2655	1772	15568	6780	8822
8	2511	3654	2470	2159	14191	6144	7962
9	3349	4419	2006	1064	19016	6371	10309
10	2949	4136	2683	1911	16560	6782	9080
11	3587	4653	2812	1954	17335	6322	9234
12	3137	4909	2667	1823	20524	8372	11525
13	3104	4494	2831	2150	16315	6969	9197
14	3180	4213	2512	1950	15172	6904	8561
15	2993	3694	1581	1718	17378	6400	9269
16	4216	5367	2915	2332	17764	7812	9885
17	2584	3780	2493	2140	15157	6389	8086
18	3750	4864	3232	2453	13782	7777	8559
19	2875	4477	2961	2062	15904	6881	8360
20	2772	4148	3123	2286	15184	7667	9589
21	2946	3897	2532	1802	12978	5678	6703
22	2715	4157	2748	1942	20516	7318	10486
*23^*	2649	3584	2981	2232	18552	6452	9965
24	3332	4554	3267	2250	17812	7626	9202
25	2755	4107	2513	1918	17095	6295	8964
*26^*	2157	3764	2638	1824	17870	7081	10770
27	2506	4056	2832	2023	16519	6637	9012
28	3538	4957	2924	2011	20202	6161	9922
*29^*	1893	2986	2412	1546	14163	5467	8311
30	3298	4585	2505	1949	18128	6387	10133
31	2425	2813	2309	1561	12952	4620	6110
32	2733	3355	2400	1762	13390	5389	7373
33	3266	4162	2752	2136	17329	6407	9407
34	3604	4865	3017	2516	18862	7676	10577
35	3868	4818	3147	2305	19538	7137	10762
36	3435	5008	2425	2036	21479	7908	11523
37	3495	5051	3189	2483	17328	7740	9337
38	2984	4310	2901	2182	13854	7505	7240

39	3197	4209	2519	1778	16356	7201	8850
40^	1042	2635	2052	1405	14997	7022	9139
41	2794	4124	2728	2158	15476	6758	8612
42	3100	4454	2895	2035	16368	7415	8959
43^	2366	3724	2969	1774	19371	7817	10936
44	2134	4160	3009	2132	15818	6725	8116
45	2765	3980	2614	1853	15321	5769	8414
46	2307	3890	2993	2296	15671	7146	8511
47	3372	4488	2630	1852	13632	6755	8335
48^	3389	4650	3140	2482	21052	8074	11924
49	3869	5542	3122	2292	21369	8072	11401
50	3149	4781	2527	1789	19146	6633	9808
51	3465	4749	3105	2204	18006	7091	9509
52^	2206	3302	2822	2177	15745	7116	8940
53	2755	4385	2769	1994	17171	8334	9749
54	2711	3955	2954	2259	14901	6393	7124
55^	1547	2621	2498	1870	15344	5597	7715
56	2859	4251	2938	2209	15795	6712	8137
57	2655	3646	1496	1414	18897	6630	10610
58	3155	4500	2527	1911	16014	6742	8820
59^	1721	2446	2096	1783	13266	5589	7601
60	2865	4043	2746	1786	16674	5777	8559
61^	2685	3408	2585	1737	18057	5834	9382
62	2785	4029	2452	1736	14668	6586	7574
63	2681	3585	2654	1938	15420	5520	8570
64	2754	3476	2667	1919	13407	6270	7489
65	3044	4289	2864	2026	14730	7181	8603
66	2336	3615	2261	1769	12321	6019	6613
67	2769	4026	2602	1920	15236	6495	8520
68	2970	3795	2138	1748	15585	8010	9930
69^	2228	3274	2618	2012	18032	7103	10939
70	3917	5135	2962	2077	19944	7462	10806
71^	4668	6727	3014	2279	25307	9599	15300
72	3197	4328	2834	2032	15274	6997	8218
73	2793	3663	2384	1973	13338	6181	7681
74	2730	3552	2647	2038	15276	6317	8589
75^	2558	3525	2882	1951	17649	6732	10705
76^	3092	3749	3136	2311	21669	6343	11529
77^	2790	4234	2606	1953	17519	6939	10069

^: Volumes extracted from T1 scan.

Table S3. Elastic Net-Selected Features (Absolute unstandardized correlation coefficients (B) descending ranked)

	Concurrent NE		*Prospective NE*		*Concurrent PE*		*Prospective PE*	
	Variables	B	Variables	B	Variables	B	Variables	B
1st-order Anatomical	SFC	1.5163	lOFC	0.9375	cACC	2.1056	mOFC	2.1724
	cACC	1.4203	SFC	0.5628	vlPFC	1.0049	lOFC	1.2130
	vlPFC	0.6636	mOFC	0.4753	rACC	0.9216	rACC	1.0217
	mOFC	0.5893	rACC	0.3624	mOFC	0.4730	SFC	1.0194
	rACC	0.4199	dlPFC	0.2450	SFC	0.4020	dlPFC	0.9970
	Imaging Modality	0.0808	Imaging Modality	-0.0172			Imaging Modality	0.3724
	lOFC	0.0699					vlPFC	0.3203
							cACC	0.3080
1st-order Sociodemographic / Clinical	3m STAI Trait Anxiety	0.8776	9m Age (wk)	1.2250	Caregiver Age	1.3551	Caregiver Age	2.3278
	3m Biological Sex	0.6038	Caregiver Age	0.8516	3m Biological Sex	1.2745	3m Age (wk)	2.0810
	Caregiver Age	0.2218	3m IBQ NE	0.6851	3m Age (wk)	0.8039	3m IBQ PE	1.9020
	3m Age (wk)	0.1572	3m PAI-BOR	0.6296	3m EPDS	-0.2777	9m EPDS	1.3300
	3m PAI-BOR	0.0983	9m STAI State Anxiety	0.5704	3m PAI-BOR	0.0739	3m STAI Trait Anxiety	0.6740
			3m Biological Sex	0.2904			9m Age (wk)	0.6283
			3m STAI Trait Anxiety	0.1200			Total Government Assistance Sum	0.4704
			9m STAI Trait Anxiety	0.1088			3m EPDS	0.2703
							3m Biological Sex	0.2053
2nd-order Anatomical x Sociodemographic Interaction	rACC x 3m Biological Sex	-0.7244	dlPFC x 3m Biological Sex	-0.5835	rACC x 3m Biological Sex	-2.1947	mOFC x Total Government Assistance Sum	1.6722
	dlPFC x 3m Biological Sex	-0.5110	mOFC x 3m PAI-BOR	0.4978	mOFC x 3m Biological Sex	1.4831	lOFC x 3m Age (wk)	-1.0205
	lOFC x 3m EPDS	-0.3414	mOFC x 9m EPDS	-0.3731	SFC x 3m STAI Trait Anxiety	1.1709	lOFC x Caregiver Age	-1.0139
	cACC x Total Government Assistance Sum	-0.1004	vlPFC x Total Government Assistance Sum	-0.3607	dlPFC x 3m Biological Sex	-0.8942	mOFC x Caregiver Age	-0.9063
	rACC x Total Government Assistance Sum	-0.0006	rACC x 9m PAI-BOR	-0.3350	mOFC x Total Government Assistance Sum	0.8020	rACC x 9m Age (wk)	-0.7028
			lOFC x 3m Biological Sex	0.3166	vlPFC x 3m Age (wk)	0.7973	lOFC x 3m EPDS	-0.6212

	lOFC x 3m PAI-BOR	0.2570	dlPFC x Caregiver Age	-0.6993	lOFC x 3m STAI State Anxiety	-0.6111
	mOFC x 3m STAI State Anxiety	-0.2458	vlPFC x 3m Biological Sex	-0.6269	rACC x 9m PAI-BOR	-0.6100
	lOFC x Total Government Assistance Sum	0.2192	SFC x 3m STAI State Anxiety	0.5863	vlPFC x 3m STAI State Anxiety	-0.5630
	dlPFC x 3m IBQ NE	0.2099	vlPFC x 3m STAI State Anxiety	-0.4976	mOFC x 3m Age (wk)	-0.5606
	SFC x 3m IBQ NE	0.2019	rACC x 3m Age (wk)	-0.4429	SFC x 3m Age (wk)	-0.5307
	vlPFC x 3m EPDS	-0.1950	dlPFC x 3m PAI-BOR	-0.3624	dlPFC x Total Government Assistance Sum	-0.5235
	rACC x 3m STAI State Anxiety	-0.1880	dlPFC x 3m EPDS	-0.2994	vlPFC x Total Government Assistance Sum	-0.4855
	mOFC x Total Government Assistance Sum	0.1833	cACC x 3m STAI State Anxiety	-0.2765	SFC x 9m Age (wk)	-0.4729
	SFC x 3m Age (wk)	-0.1734	SFC x Caregiver Age	-0.2577	lOFC x 9m EPDS	-0.4648
	lOFC x 3m IBQ NE	0.1705	cACC x Total Government Assistance Sum	-0.2339	mOFC x 3m STAI State Anxiety	-0.4533
	mOFC x 3m Biological Sex	0.1546	vlPFC x 3m EPDS	-0.2174	SFC x Total Government Assistance Sum	-0.4472
	rACC x 3m EPDS	-0.1206	SFC x 3m PAI-BOR	-0.1359	lOFC x Total Government Assistance Sum	0.4371
	SFC x 3m Biological Sex	-0.1169	cACC x 3m Age (wk)	-0.1348	rACC x 3m Biological Sex	-0.4230
	vlPFC x 3m Age (wk)	-0.0923	cACC x 3m Biological Sex	-0.0740	rACC x 9m STAI State Anxiety	-0.4212
	SFC x 9m STAI State Anxiety	0.0897	lOFC x 3m EPDS	-0.0508	vlPFC x 3m EPDS	-0.3881
	dlPFC x 3m Age (wk)	-0.0524	cACC x Caregiver Age	0.0487	rACC x 9m STAI Trait Anxiety	-0.3700
	cACC x 3m IBQ NE	-0.0505	lOFC x 3m Biological Sex	0.0256	dlPFC x 3m Age (wk)	-0.3302
	rACC x 9m Age (wk)	0.0225	mOFC x 3m EPDS	-0.0186	dlPFC x 9m STAI Trait Anxiety	-0.3262
	SFC x 3m PAI-BOR	0.0222			cACC x 3m IBQ PE	-0.3081
	rACC x 3m Biological Sex	0.0125			vlPFC x 3m PAI-BOR	-0.2721
	mOFC x 3m Age (wk)	-0.0108			SFC x 3m STAI State Anxiety	-0.2683
	lOFC x 9m Age (wk)	0.0056			lOFC x 9m Age (wk)	-0.2257
					vlPFC x 3m Age (wk)	-0.2126
					cACC x 3m Biological Sex	-0.1922
					dlPFC x 9m PAI-BOR	-0.1895
					vlPFC x 3m STAI Trait Anxiety	-0.1687
					vlPFC x 9m PAI-BOR	-0.1654
					rACC x 3m STAI State Anxiety	-0.1621
					rACC x 3m Age (wk)	-0.1554

lOFC x 9m STAI State Anxiety	-0.1526
rACC x Caregiver Age	-0.1291
vlPFC x 9m STAI State Anxiety	-0.1119
dlPFC x 3m PAI-BOR	-0.0997
dlPFC x 3m IBQ PE	-0.0997
lOFC x 3m Biological Sex	-0.0923
cACC x 3m EPDS	0.0837
cACC x 9m Age (wk)	-0.0682
dlPFC x 9m Age (wk)	-0.0501
mOFC x 3m PAI-BOR	0.0429
cACC x 9m EPDS	0.0399
mOFC x 9m STAI State Anxiety	-0.0282
vlPFC x Caregiver Age	-0.0200
lOFC x 3m IBQ PE	-0.0108
vlPFC x 9m STAI Trait Anxiety	-0.0072

Table S4. Features of Multivariate Models (Significance (p<0.05) ascending ranked)

	Concurrent NE			*Prospective NE*		
		β	p		β	p
1st-order Anatomical	cACC	0.533	0.000	mOFC	0.257	0.007
	SFC	0.402	0.000	rACC	0.193	0.016
1st-order Sociodemographic / Clinical	3m Sex	0.474	0.001	3m PAI-BOR	0.165	0.002
	3m Trait	0.112	0.004	Caregiver Age	0.219	0.007
2nd-order Anatomical x Sociodemographic Interaction	dlPFC x Sex	-0.246	0.001	dlPFC x 3m NE	0.240	0.000
	rACC x Sex	-0.259	0.013			

	Concurrent PE			*Prospective PE*		
		β	p		β	p
1st-order Anatomical	cACC	0.477	0.000	dlPFC	0.536	0.000
	vlPFC	0.396	0.000	cACC	0.398	0.000
				rACC	0.219	0.023
1st-order Sociodemographic / Clinical	3m Age	0.496	0.001	3m PE	0.902	0.000
	3m PAI-BOR	0.375	0.002			
2nd-order Anatomical x Sociodemographic Interaction	SFC x 3m State	0.465	0.000	dlPFC x 3m PE	-0.470	0.000
	vlPFC x 3m State	-0.448	0.000	cACC x 3m PE	-0.425	0.000
	SFC x 3m PAI-BOR	-0.408	0.001	vlPFC x 3m PAI-BOR	-0.084	0.023
	rACC x 3m Age	-0.385	0.009	rACC x Caregiver Age	-0.106	0.047

Additional Information

Below is additional information not central to the paper but it might be helpful to readers.

3-to-9-month Infant Emotionality Development

The 3-month NE was correlated with 9-month NE (Figure A; r^2=0.157, p=0.0078); the 3-month PE was correlated with 9-month PE (Figure B; r^2=0.255, p=0.0005). The range of changes from 3 months to 9 months were 0.24±0.72 in NE and 1.39±1.03 in PE.

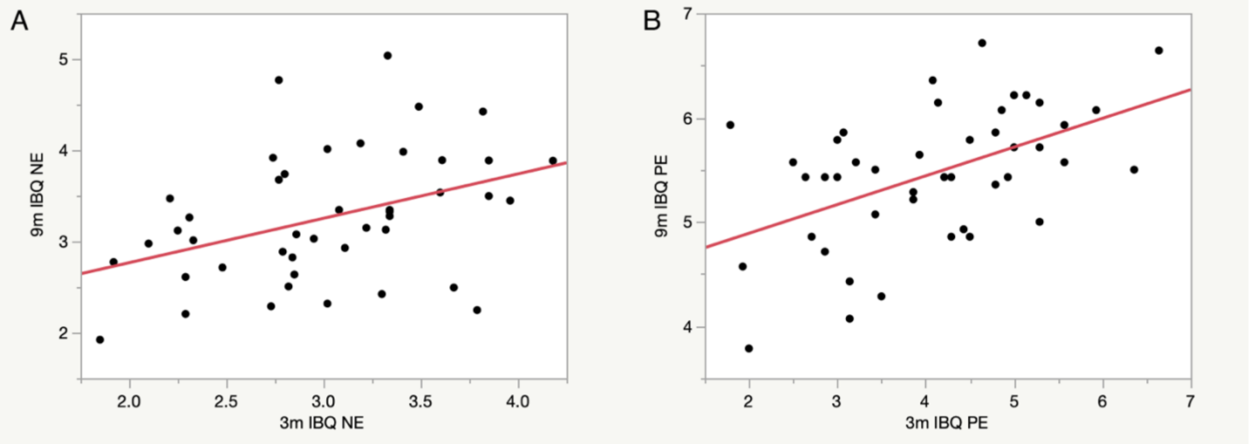

T2-only Single-modality Modeling Accuracies

For the multivariate models, the T2-only single-modality modeling accuracies were: NE a) concurrent NE T2 RMSE=0.5825; b) prospective NE T2 RMSE=0.5557; PE a) concurrent PE T2 RMSE=1.0926; b) prospective PE T2 RMSE=0.7076.

For the bivariate models, the statistics for the T2-only single-modality model for the SFC and concurrent NE correlation were β=0.283, p=0.036; for the vlPFC and prospective PE correlation were (a) β=-0.237, p=0.184, corrected by 3-month covariates; (b) β=-0.375, p=0.032, corrected by 3-month covariates with 3-month PE; (c) β=-0.373, p=0.033, corrected by 3- and 9-month covariates with 3-month PE.

Infant Factor-only Models

We also modeled the relationships between infant PFC subregional GM volumes and infant NE or PE with infant-only covariates. We summarized the RMSE (multivariate) and correlation coefficient (bivariate) of each outcome modeled with PFC subregional volumes and infant-only factors and compared them with our original models in table below. The results of these new analyses showed similarity to our original models with caregiver factors, indicating that PFC

subregional GM volumes can be used as neural markers for infant emotion development even in the absence of caregiver factors. However, given that caregiver factors critically shape infant brain development and infant emotionality, we chose to include them in our models. Moreover, we used multiple layers of feature-selection to minimize potential overfitting when more independent variables, i.e., caregiver factors, were included. We also note that selected significant features of our multivariate models all included caregiver factors, indicating caregiver factors might suppress other non-caregiver factors when modeling infant emotional behavioral outcomes.

	Concurrent NE	*Prospective NE*	*Concurrent PE*	*Prospective PE*
Multivariate Model RMSE				
Primary				
Original	0.5685	0.6111	1.0755	0.6319
Without Caregiver Factors	0.5818	0.5429	1.1678	0.6797
Replication				
Original T1/T2	0.8262/0.9416	0.8836/0.7863	1.8474/1.5332	1.3103/1.0822
Without Caregiver Factors T1/T2	0.8203/0.9529	0.7691/1.0914	1.6651/1.7125	1.0552/0.7746
Bivariate Model Correlation Coefficients	SFC			vlPFC
Primary				
Original	0.254	--	--	(a) -0.329; (b) -0.465; (c) -0.419
Without Caregiver Factors	0.280	--	--	(a) -0.215; (b) -0.378; (c) -0.377
Replication				
Original T1/T2	0.097	--	--	(a) -0.419; (b) -0.387; (c) -0.314
Without Caregiver Factors T1/T2	0.126	--	--	(a) -0.399; (b) -0.326; (c) -0.339
